# Recurrence rates for surgically treated insertional Achilles tendinopathy

**DOI:** 10.1007/s00402-023-05155-8

**Published:** 2023-12-12

**Authors:** Hubert Hörterer, Sonja Oppelt, Norbert Harrasser, Oliver Gottschalk, Wolfgang Böcker, Hans Polzer, Markus Walther, Sebastian Felix Baumbach

**Affiliations:** 1 Center for Foot and Ankle Surgery, Schön Klinik München Harlaching, Munich, Germany; 2 Department of Orthopaedics and Trauma Surgery, Musculoskeletal University Center Munich (MUM), University Hospital, LMU Munich, Munich, Germany; 3 Clinic of Orthopaedics, Klinikum Rechts der Isar, Technical University Munich, Munich, Germany; 4 Department of Orthopedics and Orthopedic Surgery, Julius-Maximilians-University, Würzburg, Germany

**Keywords:** Achilles tendon, Haglund, Insertional Achilles tendinopathy, Recurrence rate

## Abstract

**Introduction:**

Insertional Achilles tendinopathy (IAT) is a challenge for every orthopedic surgeon. Although surgical intervention is difficult to avoid after frustrating conservative therapy, little is known about recurrence of this disorder. Therefore the aim of the study was to assess the recurrence rate after primary IAT surgery.

**Materials and methods:**

The authors previous published cohort on primary IAT surgery was reanalyzed. Patients with a follow-up Foot and Function Index (FFI) at one year and final follow-up were included. IAT recurrence was defined as a FFI worsening between one year and final follow-up of > 6.5 points. General demographics, surgical details, complications, and the patient reported outcome (PROM) were assessed.

**Results:**

Out of 58 included patients (51 ± 14 years), 8 patients (14%) suffered IAT recurrence after an average of 50 ± 25 months. None of the assessed factors was predictive for an IAT recurrence.

**Conclusion:**

IAT recurrence after primary surgery occurs in up to 14% of patients.

**Level of evidence:**

IV, retrospective cohort study.

## Introduction

Insertional tendinopathy of the achilles tendon (IAT) has a reported lifetime risk of 4% to 18%, raising to up to 50% among runners [1]. Patients present with a triad of pain, swelling at the insertion of the Achilles tendon and impaired performance [2]. The treatment in general follows a clear algorithm. Conservative treatment should at least include eccentric exercises [3, 4] and extracorporeal shockwave therapy [3–5]. Still, up to one third of patients remain symptomatic [6]. Following failed conservative treatment over a period of 3 to 6 months surgery must be considered [7]. The most commonly applied surgical procedure is a midline incision, trans-achillary approach (MITA) with debridement of all pathologies present [8]. In previous studies, the authors have assessed the outcome of a uniform, single-center cohort of patients treated surgically using a MITA approach for an IAT. Although, more than 90% of the patients report a significant postoperative pain relief, about 50% of the patients suffer from residual impairments [9, 10]. One major limitation of all studies reporting on the patient rated outcome of surgically treated IAT is, that the outcome is only assessed at one point after surgery, for example after 1 or 2 years. Therefore, we are missing longitudinal data on the further course of those patients that present with residual impairments at that single time point in the follow-up. Consequently, we have no idea whether this residual impairment might develop into a recurrence of the symptoms.

In the clinical routine, orthopedic surgeons are required to inform the patients not only about the risk of a surgery, but also about the expected outcome. Both, the risk of a procedure and the expected outcome, are essential prerequisites for the patients to make an actual informed consent. But up to date, we fall short of providing the patients with valid data on the risk of a recurrence following surgical treatment of an IAT.

Therefore, the aim of the presented study was the identification of the recurrence rate of surgically treated IAT and possible influencing factors, based on a large, previously published, cohort [9, 10].

## Materials and methods

The data presented herein originate from the authors’ retrospective IAT database, which has been outlined in detail before (local ethic committee approval #17-804) [9].

The herein conducted analysis is based on the authors’ previously published patient cohort [10]. The database comprises of all patients that were treated surgically for a primary IAT following a standardized surgical procedure at a single reference center between 01.01.2010 and 01.10.2016 [9].

The inclusion criteria were age ≥ 18 years; surgically treated, isolated, unilateral IAT with initially failed nonoperative treatment of ≥ 6 months; a follow-up of ≥ 24 months; primary inhouse surgical treatment of all pathologies through a midline incision by a trans-achillary approach.

Exclusion criteria were any other surgical approaches but a midline incision, trans-achillary approach (MITA), previous surgeries to the Achilles tendon, bilateral complaints at the insertion of the achilles tendon, midportion tendinopathy of the Achilles tendon, other tendinopathies, rheumatological diseases, immunosuppressive therapy, pregnancy, other complaints causing foot and ankle disorders, or missing informed consent.

The standardized surgical procedure comprised of a midline incision, transachillary approach with debridement of all pathologies present, in each compartment of the insertion of the Achilles tendon, as recommended by van Dyk [9, 11, 12]. In case the Achilles tendon was detached more than 50% of its insertion, it was fixed either by transosseous sutures or suture anchor(s) [9, 11]. The postoperative protocol for patients with a detachment of ≥ 50% comprised of an equinus immobilization for four weeks and a stepwise reduction to neutral over another 4 weeks. If the Achilles tendon was detached < 50%, the foot was immobilized in a walker for 6 weeks in neutral position. All patients were allowed to conduct pain dependent weight bearing at week 3.

At our reference center, the patient rated outcome is routinely assessed pre-operatively and at one year follow-up, on a voluntary basis. The score used is the Foot Function Index (FFI) [13]. For the authors’ database, a further, current follow-up was assessed, again using the FFI (among others).

To analyze the recurrence rate, the authors identified all patients within this database with a preoperative FFI score and a longitudinal follow-up. A longitudinal follow-up was defined as a 1 year and final follow-up.

Recurrence was defined as a dynamic process of worsening above the minimal clinically important difference (MCID) between one year and final follow-up (4.1 years). This was done, as previous studies were able to show a rehabilitation potential for at least 12 months [8]. The MCID for the FFI has only been reported for plantar fasciitis, i.e., 6.5 points in the FFI overall [14]. This cut-off value was used herein as well. Any patient of the initial cohort, with a FFI worsening of more than 6.5 points between 1 year and final-follow-up was defined as an IAT recurrence.

Patients with an IAT recurrence were identified and compared to patients with no IAT recurrence to identify factors predisposing for IAT recurrence after surgical treatment (Table 1).

**Table 1:** Patient characteristics and surgical procedures for the primary cohort without and with recurrence of IAT after primary surgery

	Overall *n* = 58	Primary cohort* no recurrence *n* = 50	Primary cohort^*^ with recurrence *n* = 8	*p* value
Age	51 ± 14	53 ± 13	42 ± 14	0.047
Sex (% female)	41%	40%	50%	0.706
BMI	28 ± 5	28 ± 5	26 ± 3	0.231
ASA	1.6 ± 1	1.7 ± 0.5	1.5 ± 0.5	0.439
Smoking (% smoker)	9%	6%	25%	0.136
DM (% DM)	5%	6%	0%	1.0
aHT (%aHT)	31%	32%	25%	1.0
Resection posterosuperior calcaneal prominence (Haglund’s exostosis)	97%	98%	88%	0.259
Resection dorsal spur	57%	58%	50%	0.715
Resection intratendinous calcifications	35%	40%	0%	0.041
Debridement Achilles tendon	72%	70%	88%	0.423
Detachment Achilles tendon > 50%	33%	35%	14%	0.530
FHL Transfer	0%	0%	0%	1.0
Mean number of surgical procedures	3.9 ± 1.1	4.1 ± 1.2	3.1 ± 0.6	0.030
Minor complications	19%	14%	50%	0.035
Shoe conflict	19%	12%	63%	0.005

### Data assessment and analysis

First, all patients with an IAT recurrence were identified. These patients were compared to those patients with no IAT recurrence to identify factors predisposing for IAT recurrence after surgical treatment. Assessed were demographics (age, sex, BMI), medical history (smoking, diabetes mellitus, arterial hypertension), and surgical details (resection posterosuperior calcaneal prominence (Haglund’s exostosis), resection dorsal spur, resection intratendinous calcifications, debridement Achilles tendon, detachment Achilles tendon > 50%. FHL Transfer, mean number of surgical procedures).

### Statistics

The FFI scores revealed a normal distribution using the Shapiro–Wilk Test (*p* = 0.172 to *p* = 0.069). Therefore, parametric statistics were applied, and all values are given as mean ± SD, if not stated differently. The statistics applied were standard descriptives, paired and unpaired, two-tailed students *t* tests and the Chi-squared tests (Fischer exact test), where appropriate. The level of significance for the FFI, with its three subscales, was adapted per a Bonferroni alpha-level correction to *p* < 0.017. For all further analysis, a Bonferroni alpha-level correction set the level of significance to *p* < 0.004.

## Results

**Fig. 1:**
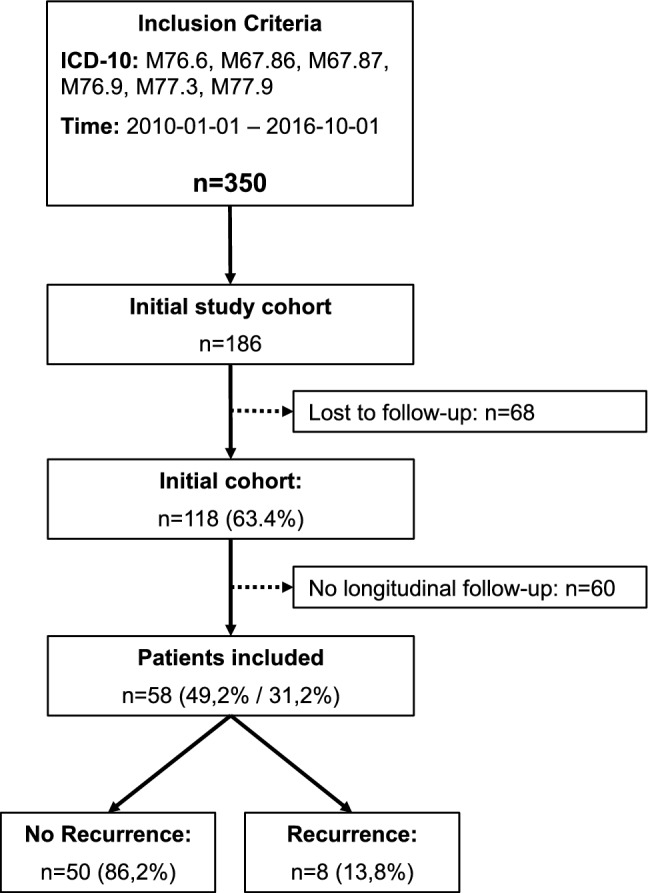
Flow-chart illustrating the patient selection for the “No Recurrence” and “Recurrence” rate cohort. After identification of the patients using ICD-10 and application of the inclusion and exclusion criteria, 58 patients were enrolled in the cohort. By the authors’ definition, 8 patients (13.8%) revealed a IAT recurrence

The overall patient selection is outlined in Fig. 1. Out of the 118 patients included in the initial publication [10], 60 were excluded from further analysis due to a missing longitudinal FFI follow-up. The longitudinal outcome of the remaining, eligible 58 patients was analyzed for recurrence, based on the above outline criteria. 86% of the patients showed no signs of recurrence, 14% had a FFI worsening of more than 6.5 points between 1 year and final follow-up and were therefore classified as recurrence cases. The final follow-up period was 50 ± 25 months and did not vary between the two groups (52 ± 25 vs. 35 ± 21 months; *p* = 0.064).

**Fig. 2:**
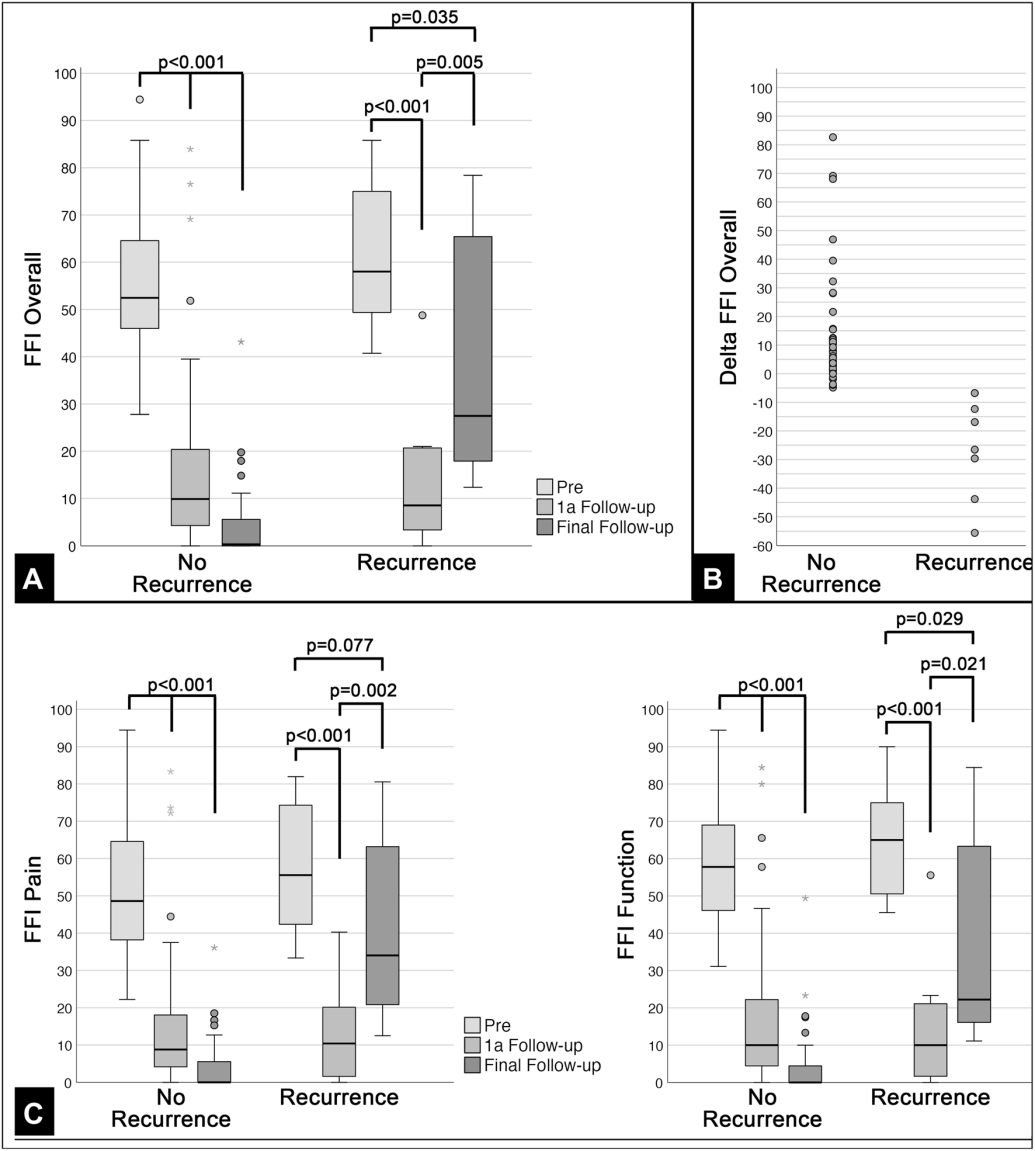
Boxplot of the FFI pre-, 1a after primary surgery, and at final follow-up (**A**) and FFI Differences between 1 year and final follow-up (**B**). The FFI subscales Pain and Function pre-, 1a after primary surgery, and at final follow-up are outlined under (**C**)

The longitudinal FFI scores for the “No Recurrence” and “Recurrence” groups are outlined in Fig. 2. For the “No Recurrence” group, the FFI Overall improved from preoperative 54 ± 15 points to 16 ± 20 points after 1 year and 5 ± 7 points at final follow-up. For the “Recurrence” group the FFI Overall improved from preoperative 61 ± 16 points to 14 ± 16 after 1 year and worsened to 39 ± 27 points at final follow-up. A pair-wise comparison revealed significant differences between each time point for the “No Recurrence” group (*p* < 0.001). For the “Recurrence” group, the FFI Overall decreased significantly between pre-operative and 1 year follow-up *(p* < 0.001) and increased significantly between 1 year and final follow-up (*p* = 0.005) (Fig. 2A). At final follow-up, the FFI Overall values did not differ significantly to the pre-operative values (*p* = 0.035). The FFI Overall difference between final follow-up and 1 year follow-up is outlined in Fig. 2B. Similar trends were found for the FFI subscales Pain and Function (Fig. 2C).

To assess factors predisposing to an IAT recurrence, both groups, the “No Recurrence” and “Recurrence, were compared in terms of demographic, medical history, and surgical details (Table 1). However, none of the evaluated parameters showed a significant influence to trigger an IAT recurrence.

## Discussion

The aim of the study was to identify the IAT recurrence rate of a previously published, surgically treated IAT cohort. The herein estimated recurrence rate of surgically treated IAT was 14%. None of the assessed parameter was predictive for an IAT recurrence.

A major hurdle in reporting recurrence rates in IAT is a missing, uniform definition of recurrence. In the current paper, recurrence was defined as a worsening of the FFI Overall score above the MCID between 1 year and final follow-up. This was done, as recurrence was considered as a dynamic process, i.e., a constant worsening over time. Per this definition, 14% of patients suffered a recurrence. Still, the chosen cut-off value is debatable. In order to put this figure into perspective, the authors applied two different approaches to define recurrence. One alternative definition could be a FFI Overall worsening above the four-fold MCID. The four-fold MCID shifts the cut-off to 26 points. This would result in a recurrence rate of 7% in the current cohort. A second approach could the upper limited of the 95% CI of FFI Overall scores. The upper 95% CI was > 14 points. Using this cut-off, 17% of the current cohort would have classified as a “Recurrence”. Consequently, one could argue that the recurrence rate could range, depending on the definition, between 7 and 17%.

Still, as recurrence remains to a certain degree subjective, the definition of cut-off values per a patient rated outcome score might be limited. An alternative approach could be the definition of recurrence as the individual decision to revision surgery. None of the herein included patients had undergone revision surgery at our institution or indicated a revision surgery when the final follow-up was collected. Still, this rigorous definition might underestimate the actual recurrence rate. One rational could be the patient’s fear of another surgery due to their disappointing first surgical experience. Secondly, the authors’ cannot preclude, that a possible revision was conducted at another institution. Consequently, although this is the first study reporting on recurrence rates for surgical treated IAT, the definition of recurrence remains debatable. Based on these considerations, the recurrence rate for surgically treated IAT apparently ranges between 7 and 17%.

The herein presented cohort is a subcohort (*n* = 58) of a previously published, larger cohort (*n* = 118). [10] In the initial study, BMI and a postoperative shoe conflict were the only factors negatively influencing the outcome of primary IAT surgery. In the current subgroup analysis, a postoperative shoe conflict was just short of becoming significant (63% vs. 12%; *p* = 0.005), but the BMI did not differ between those patients with and without a recurrence (28 ± 5 vs. 26 ± 3; *p* = 0.231). Consequently, it could be hypothesized, that the postoperative shoe conflict is the primary factor triggering the unfavorable outcomes in the recurrence group. Still, the definition of recurrence was based on a worsening between 1 year and final follow-up. It appears questionable, that a postoperative shoe conflict would worsen after one year. Moreover, patients suffering from a recurrence had fewer resections of intratendinous calcifications (0% vs. 40%; *p* = 0.041) with overall lesser surgical procedures (3.1 ± 0.6 vs. 4.1 ± 1.2; *p* = 0.030). In contrast, the rate of intratendinous calcifications was lower in the initial cohort (26% vs. 56%) [10]. Consequently, one could hypothesis, that those patients with a less aggressive surgical debridement were more likely to suffer a recurrence. However, previous studies questioned the pathognomic effect of intratendinous calcifications. Greiner et al., for example, did not observe a correlation between recurrent calcifications and return of symptoms [15]. A prospective observational study identified risk factors such as increasing age, BMI, diabetes, hypercholesterolemia and hypothyroidism for calcifying IAT. Furthermore, a BMI > 30 was associated with a higher incidence of calcifications. Even if these comorbidities favour the incidence of insertional calcific tendinopathy (ICT), it is debatable whether they impact for a IAT recurrence as only few patients with ICT become symptomatic [16].

Although the authors tried to account for various co-founders, such as age, sex, BMI, DM and aHT, other conditions affecting IAT were not recorded. One of the most pronounced might be rheumatologic diseases [17]. At our reference centers, patients are screened for clinical indicators for rheumatologic diseases and bilateral cases were excluded. Still, these precautions do not rule out a possible rheumatologic genesis of the IAT.

In general, the authors consider the homogeneous surgical approach a strength of the study. The surgical approach comprised of a MITA approach and debridement of all pathologies present. Therefore, the study does not allow to draw conclusions on different surgical treatment strategies. With respect to the surgical approach, Maffulli et al. were able to show a decrease for surgical side infections for the Cincinnati approach compared to other commonly applied approaches in IAT surgery [18]. Still, up to date we do not know which surgical approach to use for which combinations of pathologies in IAT.

Several limitations already have been mentioned. The major limitations are the retrospective study design and a lost-to follow-up rate of 51%. One reason could be that dissatisfied patients suffering an IAT recurrence choose another clinic or physician for further treatment after primary surgery. But previous studies on primary IAT surgery have comparable lost-to follow-rates [19, 20]. An additional flaw of the presented study is that there is no consistent longterm diagnostics. Nonetheless, past research has suggested that radiological pathology at the insertion of the Achilles tendon does not necessarily indicate the pain causing pathology [21]. This correlation may extend also to the recurrence of insertional Achilles tendinopathy. The definition of IAT recurrence is debatable, but in course of the discussion, the authors have tried to apply alternative definitions to provide a realistic idea of the actual recurrence rate. Still, this is the first study to assess the recurrence rate after primary IAT surgery in a larger cohort.

## Conclusion

Patients undergoing primary surgery for IAT must be informed, that 7–17% of patients will suffer a recurrence. The authors were not able to identify factors predisposing for a recurrence. Future studies should focus on biological factors in patients undergoing IAT surgery.
